# Urinary peptidomics analysis reveals proteases involved in diabetic nephropathy

**DOI:** 10.1038/s41598-017-15359-9

**Published:** 2017-11-09

**Authors:** Magdalena Krochmal, Georgia Kontostathi, Pedro Magalhães, Manousos Makridakis, Julie Klein, Holger Husi, Johannes Leierer, Gert Mayer, Jean-Loup Bascands, Colette Denis, Jerome Zoidakis, Petra Zürbig, Christian Delles, Joost P. Schanstra, Harald Mischak, Antonia Vlahou

**Affiliations:** 1 Biomedical Research Foundation Academy of Athens, Athens, Greece; 2 Mosaiques Diagnostics GmbH, Hannover, Germany; 3 Department of Pediatric Nephrology, Hannover Medical School, Hannover, Germany; 4 Institut National de la Santé et de la Recherche Médicale (INSERM), U1048, Institut of Cardiovascular and Metabolic Disease, Toulouse, France; 5 Université Toulouse III Paul-Sabatier, Toulouse, France; 6 Department of Diabetes and Cardiovascular Science, University of the Highlands and Islands, Centre for Health Science, Inverness, IV2 3JH UK; 7 Department of Internal Medicine IV (Nephrology and Hypertension), Medical University Innsbruck, Innsbruck, Austria; 8 Institut National de la Santé et de la Recherche Médicale (INSERM), U1188 - Université de La, Réunion, France; 9 Institute of Cardiovascular and Medical Sciences, BHF Glasgow Cardiovascular Research Centre, University of Glasgow, 126 University Place, Glasgow, G12 8TA UK

## Abstract

Mechanisms underlying the onset and progression of nephropathy in diabetic patients are not fully elucidated. Deregulation of proteolytic systems is a known path leading to disease manifestation, therefore we hypothesized that proteases aberrantly expressed in diabetic nephropathy (DN) may be involved in the generation of DN-associated peptides in urine. We compared urinary peptide profiles of DN patients (macroalbuminuric, n = 121) to diabetic patients with no evidence of DN (normoalbuminuric, n = 118). 302 sequenced, differentially expressed peptides (adjusted p-value < 0.05) were analysed with the Proteasix tool predicting proteases potentially involved in their generation. Activity change was estimated based on the change in abundance of the investigated peptides. Predictions were correlated with transcriptomics (Nephroseq) and relevant protein expression data from the literature. This analysis yielded seventeen proteases, including multiple forms of MMPs, cathepsin D and K, kallikrein 4 and proprotein convertases. The activity of MMP-2 and MMP-9, predicted to be decreased in DN, was investigated using zymography in a DN mouse model confirming the predictions. Collectively, this proof-of-concept study links urine peptidomics to molecular changes at the tissue level, building hypotheses for further investigation in DN and providing a workflow with potential applications to other diseases.

## Introduction

Diabetic nephropathy (DN) remains the leading cause of end-stage renal disease worldwide, contributing to mortality in affected patients^1,2^. Moreover, deterioration of kidney function is associated with severe cardiovascular events in diabetic patients^3^. Current diagnostic tools for DN include measurement of glomerular filtration rate (GFR) and urinary albumin excretion rate (AER), however both markers lack the ability to reliably predict disease progression^4^.

Investigation of molecular pathways affected in DN can provide new insights regarding disease pathophysiology and assessment of onset and disease progression. Intracellular maintenance of protein homeostasis has been attributed to cooperation between molecular chaperones and proteases^5^. Altered protease activation and protease-dependent signalling has been linked to hallmarks of kidney disease such as inflammation and tissue remodelling^6^. Multiple studies suggest that high glucose levels are related to inhibition of matrix metalloproteinases (MMPs), leading to accumulation of extracellular matrix (ECM) in the kidney tissue^7^. Along these lines, high activity of dipeptidyl peptidase-4 (DPP-4), a multifunctional cell surface aminopeptidase, was suggested to contribute to renal fibrosis in DN and its inhibition showed renoprotective effects in recent studies^8,9^. Moreover, pronounced hypertension induced by proteolytic activation of the epithelial sodium channel (ENaC) was found related to increased renal filtration of plasminogen (activated to plasmin in urine), prostasin and urokinase^10^. Undoubtedly, proteases play a significant role in pathogenesis of DN, however, given the complexity of proteolytic systems, further research efforts are required to gain a broader view of their function in the disease.

In the era of high-throughput technologies, increasing effort is placed on discovery and development of non-invasive biomarkers. A prominent example of such projects is the development of a urinary peptide biomarker panel, the CKD 273 classifier, for diagnosis of chronic kidney disease (CKD) progression^11^. Moving one step further, recently published studies aim at elucidating the mechanism responsible for the generation of urinary peptides and therefore identify probable biological pathways altered at the tissue level^12,13^.

The aim of this study was to predict proteases involved in the generation of urinary peptides differentially expressed in DN patients in comparison to diabetic controls exhibiting normal urinary albumin levels. Furthermore, algorithms were developed to estimate *in silico* the activity trend of the deregulated proteases. Predicted findings were investigated in the context of available kidney tissue transcriptomics data and relevant literature and, in selected cases, validated by gelatin zymography in DN mouse models. Therefore, this study provides an integrated workflow and can serve as a proof of principle for linking urinary peptidomics data to tissue events in DN, ultimately providing a path towards liquid biopsy in nephrology^14^.

## Results

### Differentially Expressed Peptides

**Figure 1:**
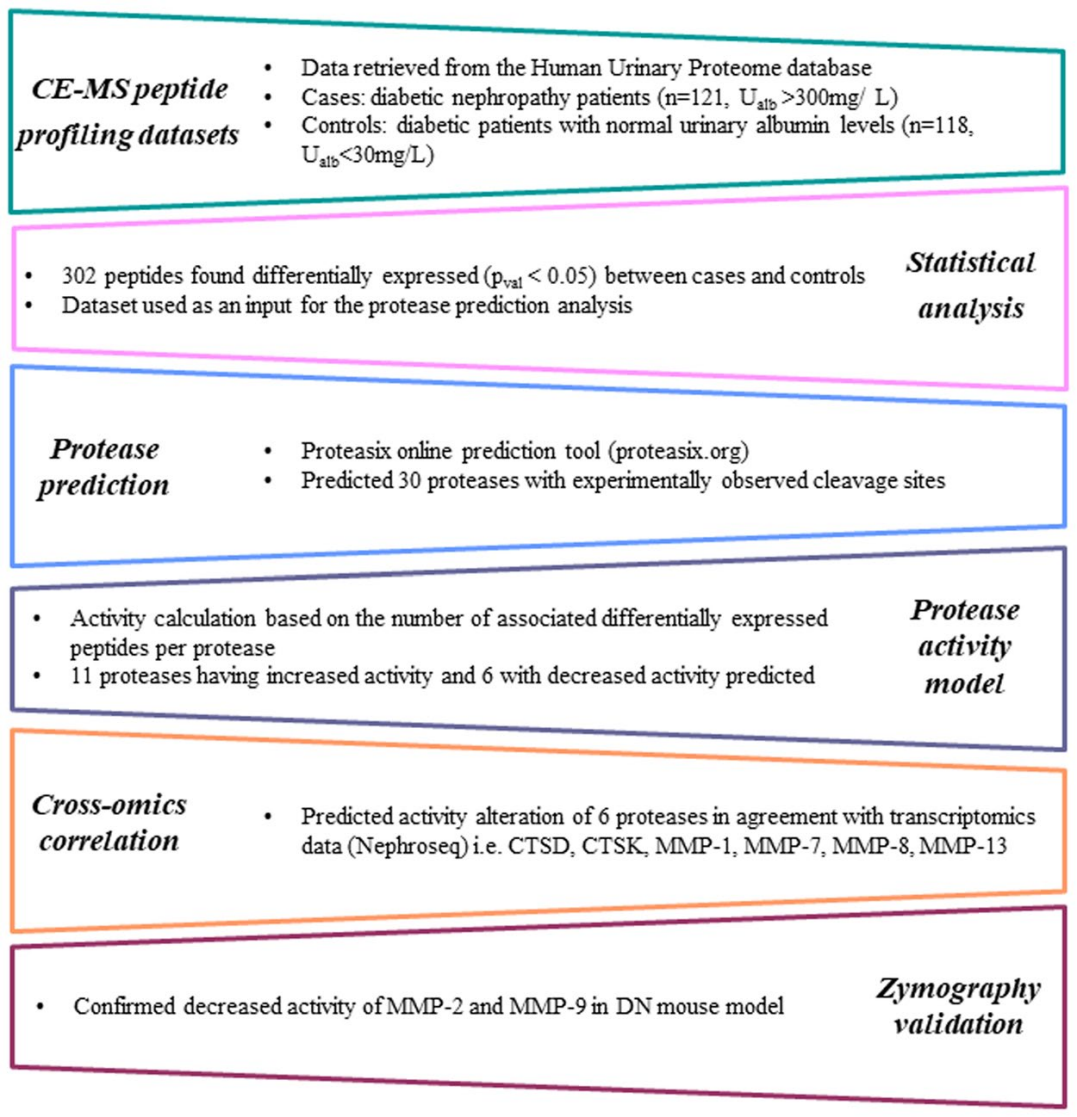
Project workflow and main results.

The main steps of the study are outlined in Fig. 1. The analysis focused on comparison of urinary peptides in diabetic patients with nephropathy (n = 121, urinary albumin levels >300 mg/L, DN) vs. patients without evidence of DN (n = 118, urinary albumin levels <30 mg/L, non-DN; Table 1). Out of a total of 1385 detected peptides, 302 peptides were found to be differentially abundant at statistically significant levels (p_val_ < 0.05, following adjustment for multiple testing) and with an over 50% change in abundance in DN urine samples in comparison to controls (Supplementary Table 1). These included multiple fragments of alpha-1 antitrypsin (41 fragments, including isoforms carrying different post-translational modifications such as peptide IDs 40409 and 38879), collagen A1 (I) (57 fragments), collagen A2 (I) (10 fragments), collagen A1 (III) (34 fragments), beta-2-microglobulin (14 fragments), but also fragments of serum albumin, various apolipoproteins (I, II, IV), fibrinogen and uromodulin (Supplementary Table 1). Of these peptides, 117 were found at lower and 185 at higher levels in DN versus non-DN group. The distribution of deregulated peptides based on protein of origin is presented in Table 2.

**Table 1:** Clinical characteristics of the patients.

	With albuminuria (Ualb >300 mg/L)	Without albuminuria (Ualb <30 mg/L)	P-value
N	121	118	—
Age	58 ± 13	58 ± 13	0.989
Male/Female	80/41	82/36	1
DM Type 1/Type 2	47/74	47/71	0.357
HbA1c	9.1 ± 2.0	7.9 ± 1.6	0.024
Urine albumin (mg/L)	880 ± 760	10.8 ± 7.5	<0.001
Creatinine (mg/dl)	1.58 ± 0.89	1.05 ± 0.36	<0.001
SBP	144 ± 20	133 ± 19	<0.001
DBP	80 ± 10	76 ± 10	0.001
eGFR (ml/min/1.73 m2)	68 ± 36	76 ± 27	0.034
LDL (mmol/L)	2.3 ± 1.0	2.3 ± 0.7	0.688
HDL (mmol/L)	1.1 ± 0.5	1.5 ± 0.5	0.497

Abbreviations: Ualb – urine albumin, SBP - Systolic Blood Pressure, DBP - Diastolic Blood Pressure, eGFR - Estimated Glomerular Filtration Rate, LDL - low-density lipoprotein, HDL - high-density lipoprotein.

**Table 2:** Distribution of deregulated peptides based on protein of origin (top 10 proteins are presented).

Corresponding protein	Total number of peptides	Upregulated peptides	Downregulated peptides
CO1A1_HUMAN	**57**	9	48
A1AT_HUMAN	**41**	41	—
CO3A1_HUMAN	**34**	14	20
B2MG_HUMAN	**14**	13	1
HBA_HUMAN	**13**	11	2
CO1A2_HUMAN	**10**	9	1
FETUA_HUMAN	**9**	9	—
ALBU_HUMAN	**9**	9	—
APOA1_HUMAN	**7**	7	—
HBB_HUMAN	**6**	5	1
UROM_HUMAN	**6**	3	3

### Protease Prediction and Activity Calculation

Protease prediction with Proteasix^15^ (www.proteasix.org) was performed applying stringent criteria (only accepting proteases with experimentally observed cleavage sites, as described in the Methods section), which yielded a list of 30 proteases putatively responsible for the generation of the 302 differentially expressed urinary peptides (Supplementary Table 2). Given the broad substrate specificity of most proteases^16^, one protease could be assigned to different peptide sequences (e.g. predicted cleavage of hemoglobin subunit alpha, hemoglobin subunit beta and osteopontin by cathepsin D) and conversely, one peptide sequence could be cleaved by different proteases (e.g. predicted cleavage of collagen alpha-2 (I) [844–865] fragment by MMP-2, MMP-13, MMP-14). For the 30 proteases, a total of 135 associated peptides (corresponding to 53 unique peptide IDs) and their regulation trend were extracted from the input list (Supplementary Table 2). An activity score was then calculated (described in the Methods section) potentially reflecting the predicted alteration in catalytic activity of each protease (Table 3, Supplementary Table 3). A threshold of a minimum of 3 associated peptides per protease was applied to increase reliability of prediction, which resulted in a final list of 17 deregulated proteases (Table 3). Among the highest scoring proteases predicted to have increased proteolytic activity are cathepsin D (CTSD), kallikrein 4 (KLK4), proprotein convertases (PCSK4, PCSK5, PCSK6 and PCSK7) and matrix metalloproteinase-7 (MMP-7). Potentially inhibited proteases included various matrix metalloproteinases i.e. MMP-13, MMP-9, MMP-2, MMP-3, MMP-8 and MMP-12. Predicted proteases together with the respective calculated activity scores are presented in Table 3.

**Table 3:** List of predicted proteases with calculated activity score.

Protease	↓pept.	↑pept.	%freq	Score	Transcriptomics expression (Nephroseq; in DN vs. controls)	Protein expression (Musante *et al*.^4^; in DN vs. diabetics)
**Activated**						
CTSD	1	9	80	**9.65**	increase	increase
KLK4		7	100	**7.53**	—	—
PCSK4		7	100	**7.53**	—	—
PCSK5		7	100	**7.53**	inconclusive*	—
PCSK6		7	100	**7.53**	decrease	—
PCSK7		7	100	**7.53**	inconclusive*	—
MMP-7		5	100	**5.38**	increase	increase
CTSK		3	100	**3.23**	increase	—
MMP-26		3	100	**3.23**	decrease	—
MMP-20	2	3	20	**1.60**	—	—
MMP-1	1	2	33	**1.51**	increase	—
**Deactivated**						
MMP-13	10	4	−43	**−12.05**	decrease	no change
MMP-9	10	6	−25	**−7.57**	—	decrease
MMP-2	4	1	−60	**−6.36**	increase	no change
MMP-3	4	1	−60	**−6.36**	increase	increase
MMP-8	5	2	−43	**−6.02**	decrease	no change
MMP-12	4	2	−33	**−3.89**	—	no change

(↓ pept.: number of downregulated peptides, ↑ pept.: number of upregulated peptides). *Inconclusive: some studies reported increased and some decreased levels of the respective protease mRNA in DN (studies are listed in Supplementary Table 4).

### Validation of Predictions

#### Cross-omics correlations in Nephroseq database

To place our predictions in the context of existing literature in the field and compile relevant evidence, as a first step, kidney transcriptomics data from DN patients and healthy controls were retrieved from the Nephroseq database (www.nephroseq.org) and the mRNA level of expression of the 17 predicted proteases in DN was evaluated. In total, 5 profiling datasets comparing DN vs. healthy control subjects were found in the database, from which protease transcripts (p_val_ < 0.05 in DN vs. controls) were extracted. Those datasets originated from 3 scientific publications related to DN i.e. Ju *et al*.^17^, Schmid *et al*.^18^ and Woroniecka *et al*.^19^ (summarized in Supplementary Table 4). We found a total of 12 protease transcripts overlapping with the 17 predicted proteases (Table 3 and Supplementary Table 5). The predicted protease activity trend was in agreement with the respective mRNA expression trend for 6 proteases, namely CTSD, CTSK, MMP-1, MMP-7, MMP-8 and MMP-13. For 4 proteases (PCSK6, MMP-2, MMP-3, MMP-26), opposing expression trends at the mRNA level could be observed in comparison to the predicted activity changes, whereas in the cases of PCSK5 and PCSK7 the transcriptomics data were inconclusive with respect to providing clear expression trends for the respective mRNAs in DN (Table 3).

#### Literature datasets

We further correlated our predictions with protein expression data on proteases of urinary extracellular vesicles (UEVs) in type 1 DN patients, as reported by Musante *et al*.^4^. In this study, authors investigated changes in the expression of 34 proteases and 32 protease inhibitors through analysis of urine samples of diabetic patients divided in normo-, micro- and macroalbuminuric groups^20^. We compared the relative protein expression change of the reported protease levels between normo- and macroalbuminuric patients with the deregulated proteases predicted in our analysis (Table 3). Out of the 4 proteases found to be significantly changing in disease in both datasets (i.e. our study and the study of Musante *et al*.^4^), the predicted deregulation of 3 (CTSD, MMP-7, MMP-9) was in agreement with the reported protein expression trend and 1 (MMP-3) presented opposite trend (Table 3). Collectively, these comparisons to the existing relevant literature did not allow reaching conclusive statements with respect to our predictions, in part due to differences in study design (different cases and controls in each case), which further enhanced the need for experimental verification of our findings.

#### Validation of predicted activity of MMP-2 and MMP-9 in DN mouse model through zymography

**Figure 2:**
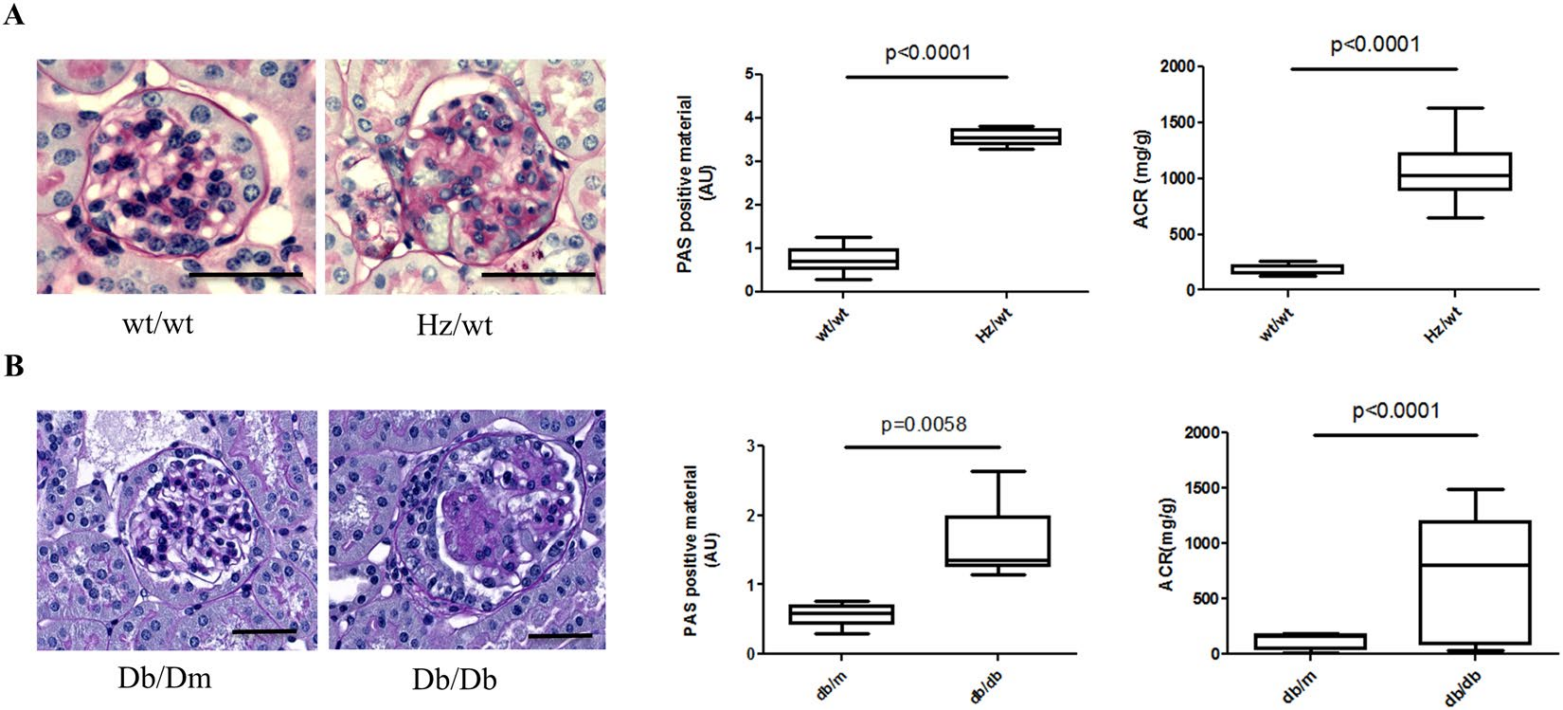
Renal lesions in DN mouse models. Both (**A**) 4 months old type 1 diabetic mice (Hz/wt) and (**B**) 24 weeks old uninephrectomized type 2 diabetic mice (Db/Db) display significantly increased glomerular PAS + material and increased urinary albumin to creatinine ratio (ACR) compared to control mice, wt/wt and Db/Dm, respectively. N = 6–10 mice. Bar 40 µm.

**Figure 3:**
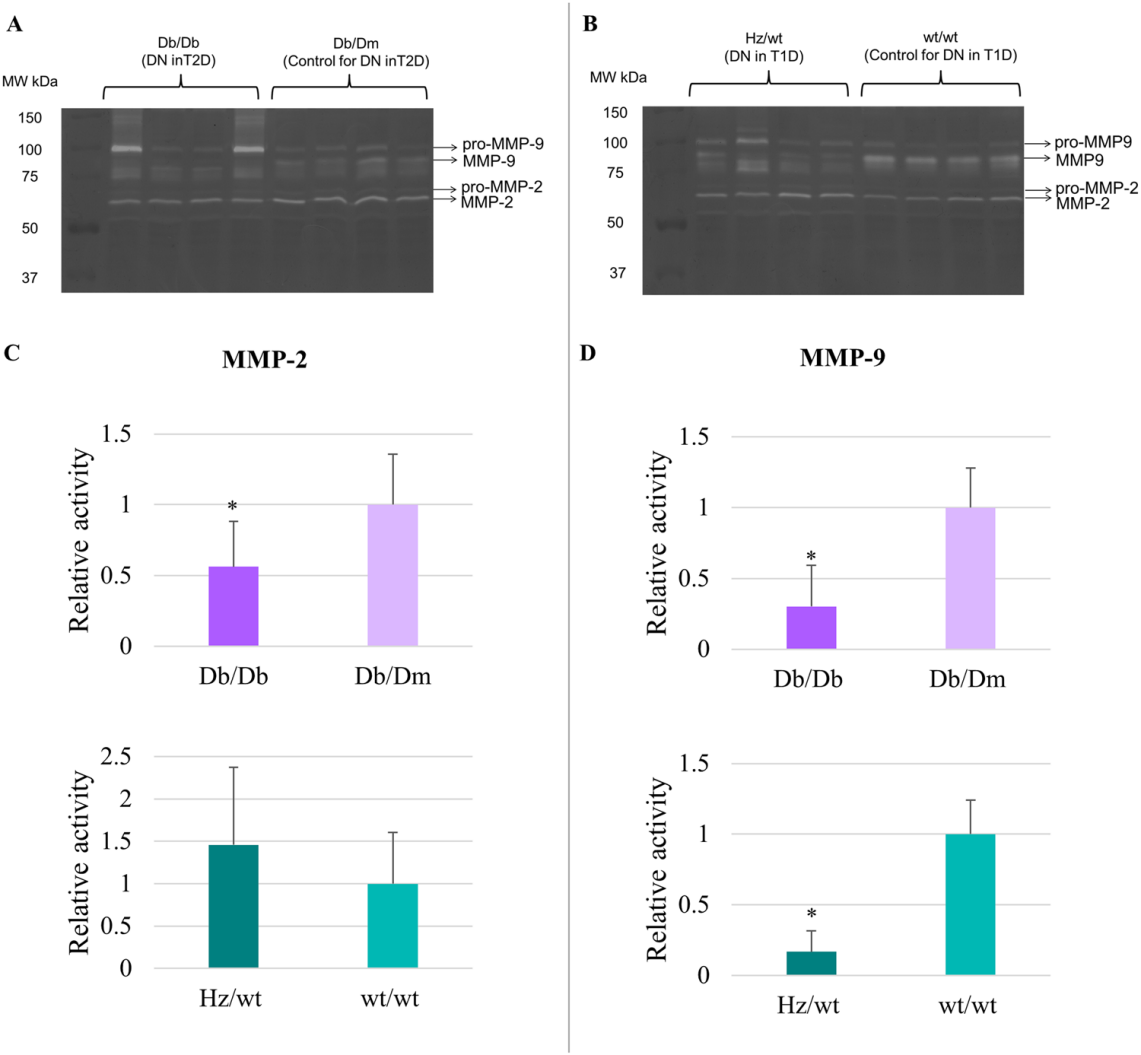
Zymography for the investigation of MMP-2 and MMP-9 activities. (**A**) Extracts from four biological replicates of DN in type 2 diabetes (T2D; Db/Db) and their respective controls (Db/Dm) were loaded. (**B**) Extracts from four biological replicates of DN in type 1 diabetes (T1D; Hz/wt) and their respective controls (wt/wt) were loaded. (**C**) The fold change of MMP-2 activity in DN was assessed relative to the respective controls per category: Db/Db (DN in T2D) was compared to Db/m and Hz/wt (DN in T1D) to wt/wt. The mean value for Db/Db vs. Db/Dm (activity fold change) was 0.56 ± 0.32 (p_val_ < 0.05), whereas the mean value for Hz/wt vs. wt/wt (activity fold change) was 1.46 ± 0.91 (p_val_ > 0.05). (**D**) The fold change of MMP-9 activity in DN was assessed relative to the appropriate controls for each group. (**A**) Db/Db (DN in T2D) was compared to Db/m and Hz/wt (DN in T1D) to wt/wt. The mean value for Db/Db vs. Db/Dm (activity fold change) was 0.30 ± 0.29 (p_val_ < 0.05), whereas the mean value for Hz/wt vs. wt/wt (activity fold change) was 0.17 ± 0.15 (p_val_ < 0.05). Graphical representation of densitometry analysis of the results (mean ± SD) is shown (∗p_val_ < 0.05, Student’s t-test).

Given the biological relevance of the proteases involved (described in the Discussion) and since no objective algorithm for prioritizing the findings was available, the generated hypotheses with respect to the activity changes in DN were selected for further experimental verification based on assay availability. Specifically, the collagenolytic profile (reflecting MMP-2 and MMP-9 activity) of kidney tissue from diabetic, both type 1 (Ins2^Akita^, Hz/wt) and 2 (Db/Db), and control mice was analysed by zymography. The mice clearly displayed DN as exemplified by the significant presence of glomerular PAS positive material and an increased albumin to creatinine ratio (ACR, Fig. 2). Representative gel images from each group are presented in Fig. 3. Zymography of kidney extracts from these mice showed that the active MMPs and their respective pro-forms were well-separated, with the pro-MMP-9 identified at 92 kDa, the active MMP-9 at 82 kDa, the pro-MMP-2 identified at 72 kDa and the active MMP-2 at 66 kDa (Fig. 3A and B - arrows). In case of mice with type 2 diabetes (Db/Db vs. Db/Dm), we report a significant 0.56 (±0.32) fold decrease of activity of active form of MMP-2 in comparison to the respective controls (Fig. 3C, p_val_ < 0.05). In contrast, MMP-2 activity was not significantly changed in type 1 DN (Hz/wt vs. wt/wt). The activity of MMP-9 decreased in both, type 1 and type 2 DN mice when comparing to the respective controls (Fig. 3D). Specifically, for MMP-9, the fold decrease of activity in Db/Db vs. Db/Dm was 0.30 (±0.29, p_val_ < 0.05), and 0.17 (±0.15, p_val_ < 0.05) in Hz/wt vs. wt/wt comparison supporting the activity predictions from the peptidomics analysis.

## Discussion

Prevalence of diabetic nephropathy is continuously increasing and to date accounts for approximately 50% of end-stage renal disease cases worldwide^21–24^, underscoring the necessity and urgency to identify the mechanisms underlying pathogenesis of DN. Proteases are involved in a variety of molecular processes and their strict regulation is necessary to maintain tissue homeostasis and prevent tissue damage^25,26^. Thus, careful evaluation of proteases in the context of DN appears highly relevant when aiming towards understanding disease pathophysiology, even of potential diagnostic or therapeutic value^27,28^. Histological features of DN, including mesangial ECM expansion, thickening of glomerular and tubular basement membrane, tubular atrophy, and interstitial fibrosis, suggest changes in proteolytic events in the kidney as directly involved in disease pathogenesis^27^. Changes in glomerular matrix proteins in DN are widely reported and include increased deposition of collagen I, III IV, V, fibronectin and entactin^29^. Nonetheless, knowledge on proteases in DN is still very limited. Through this study, we provide a proof of concept for linking urinary peptidomics data to altered protease activity in DN, highlighting several proteases, which could be possible drivers of disease progression and should be further investigated.

Matrix metalloproteinases (MMPs), collectively, are able to degrade every component of ECM and have been implicated in renal fibrosis progression^30^. Their dysregulation was already linked to kidney pathogenesis, including DN^31^. However, given the complexity of MMPs pathobiology, results described in various reports regarding their activity in DN are not always consistent^24,31^. Specifically, existing differences between protease activity reported in cell lines, animal models and human disease (e.g. in case of gelatinases, membrane-type MMPs) are being explained by potential diversity in treatment options, analysis of whole kidney versus tissue compartments and potentially differing mechanisms responsible for DN development in rodents and human^24^. In our analysis, increase in proteolytic activity of MMP-1, MMP-7, MMP-20, MMP-26 and decreased activity of MMP-2, MMP-3, MMP-8, MMP-9, MMP-12 and MMP-13 in DN were predicted.

MMP-2 and MMP-9 are unique among MMPs for preferentially degrading collagen type IV^32^ – major component of tubular basement membrane (TBM)^33^. As such, activity of these proteases seems of high importance in the context of DN development, being specifically related to hallmarks of DN including tubulointerstitial changes and the thickening of tubular basement membrane^24,34^. Activity of MMP-2 and MMP-9 in kidney tissue of diabetic patients has not been extensively investigated, however, activity assays in urine indicate their increase in diabetes^35^. In animal models of diabetic kidney disease, decrease in abundance and activity of MMP-2 and MMP-9 was reported in the early diabetes, while changes in MMP-9 activity are generally not detected in the advanced diabetes^36^. On the other hand, MMP-9 upregulation and increase in activity have been implicated in kidney fibrosis initiation through induction of epithelial–mesenchymal transition (EMT)^37–39^. In agreement with our prediction, reduced activity of MMP-2 and MMP-9 in the mouse DN model was observed (both DN type 1 and 2 for MMP-9 and DN type 2 for MMP-2). Along these lines, decreased activity of MMPs in urinary extracellular vesicles (UEVs) of DN patients in comparison to normoalbuminuric diabetic patients, was reported by Musante *et al*.^4^, leading collectively to the conclusion that kidney damage is associated with reduced activity of MMP-2 and MMP-9 in diabetic kidney tissue. Validation in human kidney tissue should be considered as a key step in the research of the role of these proteases in DN.

Possible activation of matrix metalloproteinase 7 (MMP-7) was reported in several studies (as summarized in Table 3), strengthening our prediction. MMP-7 activity in DN has not been extensively studied, however, increased tissue expression of MMP-7 has been reported in various kidney diseases (including DN)^40^. Moreover, elevated urinary levels of MMP-7 have been reported in patients with various kidney diseases compared to healthy controls and correlated well with renal fibrosis scores. Therefore, MMP-7 has been proposed as a putative urinary biomarker of kidney fibrosis in CKD and possible mediator of fibrotic events^41,42^. Along these lines, peptides that we detected and presumably originated from MMP-7 activity belong to cleaved alpha-1-antitrypsin (inhibitor of the following proteases: trypsin, chymotrypsin, and plasminogen activator) and fibrinogen alpha chain (monomers of which form the fibrotic tissue)^41^.

Proteases from the family of lysosomal cathepsins, although linked to tissue fibrosis, are not extensively studied the context of kidney disease^13^. Cathepsin D (CTSD), predicted to have increased activity based on our analysis, contributes to intracellular protein degradation and is known to mediate inflammation and atherosclerotic events^43^. Cysteine proteases i.e. cathepsin B and L take part in CTSD maturation^44^, the latter being implicated in development of early experimental DN^45^. Fox *et al*. investigated the role of CTSD in CKD, reporting its increased abundance in the human kidney tissue of CKD patients (including DN), especially in the areas of tubular damage. Additionally, activity of CTSD was increased in kidneys of two independent CKD mouse models (unilateral ureteric obstruction (UUO) and chronic ischemia reperfusion injury (IRI) vs. respective controls) and its inhibition by pepstatin A effectively reduced interstitial fibrosis^46^. Similar increase in activity in injured kidney and benefits of CTSD inhibition were reported by Cocchiaro *et al*. in the acute kidney injury (AKI) mouse model^47^. Since cathepsin D is involved in pathways related to collagen degradation and metabolism of angiotensinogen to angiotensins^48^, its increased expression and activity is expected in DN. Moreover, CTSD, which synthesis is stimulated by insulin^49^, was suggested as a novel biomarker of type 2 diabetes risk and insulin resistance (IR)^50^. Nevertheless, Musante *et al*.^4^ reported decreased activity of CTSD in exosomes of DN patients in contrast to its increased protein levels, collectively underscoring the need for further investigation of this protease and its specific role in the context of DN. Interestingly, Kusaka *et al*. reported increased serum levels of CTSK having positive correlation with nephropathy progression in diabetic patients (assessed based on serum creatinine and eGFR measurements)^51^. A 7-year long follow-up study performed by the same authors concluded that serum CTSK levels in type 2 diabetes patients are indicative of vascular endothelial dysfunction and deterioration of renal function^52^. Taken together, cathepsins D and K, both extensively studied for their potential as therapeutic targets in e.g. cancer, Alzheimer or Parkinson disease^53,54^, may have potential as targets for novel DN treatment.

In conclusion, we present a novel approach for the interpretation of changes in urinary peptides, leading to prediction of upstream proteolytic events, possibly linking the peptidome profile to tissue proteases in DN. Through our analysis, we indicated possible deregulation of several proteases in DN and subsequently verified experimentally selected results in the case of MMP-2 and MMP-9 in DN mouse models. Among the weaknesses of our study is the relatively low number of protease-associated sequences; their enrichment with more disease-associated peptides/potential substrates of these proteases will further increase confidence in the predicted protease activity trends. A further weakness is the fact that only some of the predicted changes in protease activity were experimentally verified. We are fully aware that the study of proteases is far more complex: for any given enzyme, mRNA, protein and activity levels are not always in agreement, as also evident from the data presented here. Hence, the described cross-correlations should be interpreted with caution. Moreover, substrate preference and specificity can be highly variable and complex compensatory mechanisms are activated in pathological conditions^6^. In addition, advanced glycation end products (AGE) can play a role in protease activity modulation, further increasing the intricacy^55^. Nevertheless, our analysis yielded a list of functionally interesting findings which should be further investigated in the context of DN progression and could be considered as putative therapeutic targets. Through our analysis we contribute to a better understanding of the protease role in DN and propose multiple proteases of possible significance in disease pathogenesis.

## Methods

### Patient Population and Data Collection

The urinary CE-MS peptide profiling datasets as well as the clinical data were retrieved from the Human Urinary Proteome database^56,57^. The study was approved by the ethics committee from Hannover Medical School, Germany (ID: 3116-2016), fulfilling all the requirements on the protection of the individuals participating. Details regarding samples processing, capillary electrophoresis-mass spectrometry (CE-MS) analysis, data processing and storage can be found in Supplementary Methods. In brief, urine samples were collected from 10 different centres: Steno Diabetes Center (Gentofte, Denmark; n = 63); Austin Health and Northern Health, University of Melbourne, (Melbourne, Australia; n = 44); Diabetes Centre of the Isala Clinics, (Zwolle, The Netherlands; n = 33); Hannover Medical School (Hannover, Germany; n = 33); Human Nutrition and Metabolism Research and Training Center, Karl Franzens University of Graz (Graz, Austria; n = 19); Barbara Davis Center for Childhood Diabetes, University of Colorado Denver (Denver, USA; n = 15); Charles University (Prague, Czech Republic; n = 10); University Medical Center (Groningen, The Netherlands; n = 9); RD-Néphrologie, Néphrologie Dialyse St. Guilhem (Montpellier, France; n = 9) and BHF Glasgow Cardiovascular Research Centre (Glasgow, UK; n = 4). Sample collection was performed in accordance to local ethics requirements and all individuals gave written informed consent. In all cases, spot urine samples were collected in sterile containers at the time of the study visit. One mL aliquots of unprocessed samples were stored at −80 °C prior to being processed as described previously^58^ and Supplementary Methods. Samples were frozen within 4 hours of collection. Study participants were diagnosed with type 1 or type 2 diabetes mellitus. Diabetic nephropathy was defined by increased urinary albumin excretion (UAE) in the absence of other renal diseases. In total, we evaluated urine samples from 239 patients representing diabetic patients with nephropathy (n = 121, urinary albumin levels >300 mg/L) and patients without evidence of nephropathy (n = 118, urinary albumin levels <30 mg/L). Clinicopathological characteristics of the patients from the two groups are summarized in Table 1.

### Statistical Analysis

After testing for normal distribution, continuous data were compared by the Mann-Whitney test, as this test has proven to be of superior statistical power in peptidomics datasets^59^. In order to control for the false discovery rate at 0.05, the p-values were adjusted by the Benjamini and Hochberg method^60^ implemented in the Bioconductor package *multtest* ^61^. Differential expression was based on a p-value of <0.05 after correction for multiple testing and a fold change >1.5 for upregulated or <0.66 for downregulated peptides.

### Protease Prediction and Protease Activity Prediction

In order to link urinary peptides to the proteases potentially involved in their generation, the open-source tool for protease prediction – Proteasix (www.proteasix.org)^15^ was used for the analysis. Briefly, Proteasix uses information about naturally occurring peptides i.e. corresponding protein UniProt ID and start and stop amino acid position to predict potential cleaving proteases. Proteasix retrieves information about cleavage sites (CS) from protease databases (MEROPS, BRENDA) considering also cleavage site restrictions (from ENZYME database). As a result, a list of predicted proteases is generated. Proteases labelled as “Observed” or “Observed in different substrate” (in case given cleavage site was identified using different substrate) come from the literature-curated collection of experimentally observed cleavage site associations, whereas high/medium/low probability predictions are based on calculation of the probability of cleavage based on MEROPS protease specificity matrices. In our study, in order to increase reliability of predictions, analysis was based only on proteases with observed cleavage sites (Supplementary Table 2). As an input for our analysis, the list of the 302 sequenced, differentially expressed peptides (p_val_ < 0.05 and fold change >1.5) obtained from the CE-MS analysis was used (Supplementary Table 1). List of predicted proteases is available in Supplementary Table 2.

For the protease activity prediction the following metrics were calculated:

**occ(up):** sum of all occurrences for each individual protease in the up-regulated peptides

**occ(down):** sum of all occurrences for each individual protease in the down-regulated peptides

**n(up):** total number of peptides being up-regulated

**n(down):** total number of peptides being down-regulated

$${\bf{weight}}:(\mathrm{occ}(\mathrm{down}))/(n(\mathrm{down}))+(\mathrm{occ}(\mathrm{up}))/(n(\mathrm{up}))$$

$${\bf{freq}}{\boldsymbol{ \% }}{\boldsymbol{(}}{\bf{up}}{\boldsymbol{)}}\,=(\mathrm{occ}(\mathrm{up})/n(\mathrm{total}))\ast {\rm{100}}$$

$${\bf{freq}}{\boldsymbol{ \% }}{\boldsymbol{(}}{\bf{down}}{\boldsymbol{)}}=(\mathrm{occ}(\mathrm{down})/n(\mathrm{total}))\ast {\rm{100}}$$

$${\boldsymbol{ \% }}{\bf{freq}}\,[{\boldsymbol{ \% }}{\bf{frequency}}\,{\bf{difference}}\,{\bf{ratio}}]=((\mathrm{freq} \% (\mathrm{up})-\mathrm{freq} \% (\mathrm{down}))/(\mathrm{freq} \% (\mathrm{up})+\mathrm{freq} \% (\mathrm{down})))\ast {\rm{100}}$$

$${\bf{Score}}={\boldsymbol{ \% }}\mathrm{freq}\ast {\bf{weight}}$$

Positive scores indicate potentially activated proteases, while negative scores indicate deactivation (Supplementary Table 3).

### Prediction Investigation Through Transcriptomics Data Analysis

Nephroseq (www.nephroseq.org, University of Michigan, Ann Arbor, MI) is a kidney transcriptomics data repository equipped with tools for data analysis and visualization. The list of 17 predicted proteases was uploaded in Nephroseq in the form of EntrezGene IDs and corresponding gene expression was checked in the available diabetic nephropathy datasets. DN datasets were selected through Primary Filters > Group > Diabetic nephropathy. There were five DN datasets on comparison of DN vs. Healthy Living Donor groups (Supplementary Table 4). Significantly deregulated genes (p_val_ < 0.05) appearing in those five sets were extracted and differential expression results were compared with the predicted proteolytic activity trend.

### Mice

Ins2^Akita^ mice were used as a model of type I diabetes and were initially obtained from the Jackson Laboratory (US) and further bred in the local animal facility (Toulouse) under SPF conditions. Kidney samples (~1/6th of the kidney cortex) from 4 months old mice with diabetes (Hz/wt) and wild-type (wt/wt) mice were snap frozen after sacrifice. Db/db mice were used as a model of type 2 diabetes. Db/Dm (control) and Db/Db (diabetic) mice (Jackson Laboratory (US)) were subjected to right side uninephrectomy (UNx) under anesthesia at 8 weeks of age to accelerate development of DN as previously described^62^. Uninephrectomized Db/Dm and Db/Db mice were sacrificed at 24 weeks and kidney samples (~1/6th of the kidney cortex) were snap frozen. The animal experiments were approved by the local ethics committee from Toulouse (number 122-2015-42).

### Histology and ACR measurements

Kidneys were fixed in Carnoy solution, dehydrated in alcohol, and embedded in paraffin. Two-micrometer sections were stained with periodic acid-Schiff reagent. At least 50 glomeruli, including superficial and juxtaglomerullary cortical area, were examined for each animal. The extent of glomerular damage was obtained by manual scoring on a scale from 0–4. ACR was measured as described^63^. Briefly: the concentration of creatinine in urine was measured by the colorimetric method of Jaffe according to the protocol Creatinine Assay Kit (Bio Assay Systems). The concentration of urinary albumin was measured by ELISA using the AlbuWell kit (Exocell).

### Zymography

#### Sample preparation

Frozen kidney tissue (7 to 47 mg) was resuspended in 1x PBS (Gibco), pH 7.5 and homogenized with stainless steel beads (Next Advance) in the Bullet Blender Homogenizer (Next Advance). Samples were centrifuged (16,000 × g, 10 min, RT) to remove beads and debris. Protein concentration was determined by Bradford assay (Bio Rad) in the supernatant which was stored at −80 °C until further use.

#### Zymography protocol

Zymography was performed in 8% polyacrylamide gels containing 0.2% (w/v) gelatin (Porcine gelatin, Sigma Aldrich). Five independent experiments were performed analysing 4 biological replicates per group and the obtained data were combined in order to increase the statistical power. The protein amount of samples loaded on each well was 50 μg. Reported data include the relative fold change of protease activity among each diabetic group compared to the corresponding control, from each independent experiment. Electrophoresis was performed in TGS buffer (Bio Rad) (Tris 25 mM, Glycine 192 mM, 0.1% (w/v) SDS, pH 8.3) at 5 mA per gel, at room temperature for about 4–5 hours in non-reducing conditions. After electrophoresis, the gels were incubated with the equilibration buffer (25 mM Tris – HCl pH 7.8, Bio Rad, 2.5% (v/v) Triton-X 100, AppliChem) for 1.5 hours at 4 °C (protein renaturation). Gels were then incubated with the activation buffer (25 mM Tris – HCl pH 7.8, Bio Rad, 5 mM CaCl_2_, Sigma Aldrich) for 24 h at 37 °C followed by a wash with water and fixed with a solution of 30% methanol (Fischer), 10% acetic acid (Carlo Erba) for half an hour. Staining was performed for 30 minutes with Coomassie Brilliant Blue R-250 (Fluka) at room temperature. Finally, the gels were destained with water and scanned at a GS-800 imaging densitometer (Bio Rad) in transmission mode. The images were analysed with the Quantity One software (Bio Rad).

### Data Availability

All data generated or analysed during this study are included in this published article (and its Supplementary Information files). As described above, all methods were carried out in accordance with relevant guidelines and regulations.

## Electronic supplementary material

Supplementary Tables 1–5

Supplementary Information
