# Supplementary material for: Urinary peptidomics analysis reveals proteases involved in diabetic nephropathy
Source: Sci Rep. 2017 Nov 9;7:15160. doi: 10.1038/s41598-017-15359-9 (PMC5680307; doi:10.1038/s41598-017-15359-9)
Supplement: Supplementary file 2 — Supplementary Information [file 41598_2017_15359_MOESM2_ESM.pdf]

Urinary peptidomics analysis reveals proteases involved in diabetic nephropathy

Supplementary data

Magdalena Krochmal[1], Georgia Kontostathi[1], Pedro Magalhães[2,3], Manousos Makridakis[1], Julie Klein[4,5], Holger Husi[6], Johannes Leierer[7], Gert Mayer[7], Jean-Loup Bascands[8], Colette Denis[4,5], Jerome Zoidakis[1], Petra Zürbig[2], Christian Delles[9], Joost P Schanstra[4,5], Harald Mischak[2,9], Antonia Vlahou[1*]

[1]Biomedical Research Foundation Academy of Athens, Athens, Greece

[2]Mosaiques Diagnostics GmbH, Mosaiques Diagnostics GmbH, Hannover, Germany

[3] Department of Pediatric Nephrology, Hannover Medical School, Hannover, Germany

[4]Institut National de la Santé et de la Recherche Médicale (INSERM), U1048, Institut of Cardiovascular and Metabolic Disease, Toulouse, France

[5]Université Toulouse III Paul-Sabatier, Toulouse, France

[6]Department of Diabetes and Cardiovascular Science, University of the Highlands and Islands, Centre for Health Science, Inverness IV2 3JH, UK

[7]Department of Internal Medicine IV (Nephrology and Hypertension), Medical University Innsbruck, Innsbruck, Austria

[8]Institut National de la Santé et de la Recherche Médicale (INSERM), U1188 - Université de La Réunion, France.

[9]Institute of Cardiovascular and Medical Sciences, BHF Glasgow Cardiovascular Research Centre, University of Glasgow, 126 University Place, Glasgow, G12 8TA, UK

SUPPLEMENTARY METHODS

PROCESSING OF URINE FOR PEPTIDOMICS ANALYSIS

Urine samples were processed for peptidomics analysis as previously described[1,2]. Briefly, 700 µL of urine were defrosted with the addition of 0.1% PMSF saturated in ethanol and diluted with 700 µL of a solution containing 2 M urea, 0.1 M NaCl, 10 mM NH4OH and 0.02% SDS. The mixture was then filtered through a 20 kDa MW cut-off ultra-centrifugation filter device (Sartorius Stedim UK Ltd, United Kingdom) at 2,600 × g for one hour at 4°C. A volume of 1.1 mL of the filtrate was then loaded onto a pre-equilibrated PD-10 desalting column (GE Healthcare, Sweden) and eluted using 0.01% aqueous NH4OH. The eluate was then freeze-dried and stored at 4°C prior to resuspension in HPLC-grade water to a final protein concentration of 2 mg/mL for capillary electrophoresis-mass spectrometry (CE-MS) analysis.

CAPILLARY ELECTROPHORESIS-MASS SPECTROMETRY (CE-MS) ANALYSIS AND DATA PROCESSING

CE-MS analysis was performed using a P/ACE MDQ capillary electrophoresis system (Beckman Coulter, Fullerton, USA) on line coupled to a MicroTOF MS (BrukerDaltonic, Bremen, Germany). Samples were injected hydrodynamically at 2.0 psi for 99 sec (ca. 250 nL) and separation of peptides was achieved by reverse polarity at 25 kV for the first 30 min, and with increasing pressure (up to 0.5 psi) for another 34 min. The cartridge temperature was maintained at 25°C. Running buffer contained 79:20:1 (v/v) deionized filtered (0.2 µm) water, acetonitrile and formic acid. Sheath liquid consisted of 30% 2-propanol and 0.4% formic acid in deionized filtered (0.2 µm) water. The ESI sprayer (Agilent Technologies, Palo Alto, CA, USA) was grounded, and the ion spray interface potential was set between −4 and −4.5 kV. Spectra were accumulated every 3 seconds over a range of mass-to-charge ratios from 50 to 3000. Details on accuracy, precision, selectivity, sensitivity, reproducibility, and stability of the CE-MS method can be found in[3,4]. Internal standard peptides were used for calibration, as previously described[3]. MosaiquesVisu was used to analyse the CE-MS data[5]. Peptides were initially characterized by their molecular mass, CE-migration time, and ion signal intensity (amplitude) value. Peptide sequencing was performed using Dionex Ultimate 3000 RSLS nano flow system (Dionex, Camberly,UK) or Beckman CE, coupled to an Orbitrap Velos MS instrument (Thermo Scientific,Waltham, Massachusetts, US)[6].

The obtained spectra were analysed with Proteome Discoverer 1.2 (Thermo Scientific) (with precursor mass tolerance of 5 pmm and fragment mass tolerance of 0.05 Da) and searched against UniProt human non-redundant database. Oxidation of methionine and proline were considered as variable modifications. Criteria for sequence acceptance were high confidence score (Xcorr ≥ 1.9) and lack of unmodified cysteine. To prevent false identification of sequences, a strong correlation between peptide charge at the working pH of 2 and capillary electrophoresis migration time was used[7]. All detected peptides were annotated, matched and deposited in a Microsoft SQL database (Human Urinary Proteome Database[8,9]), allowing for further analysis and comparison between case and control groups.

SUPPLEMENTARY TABLES LEGENDS

Supplementary Table 1. List of 302 sequenced, differentially expressed peptides (pval <0.05, Mann-Whitney) in comparison between diabetic patients with macroalbuminuria (Ualbumin >300 mg/L) vs. diabetic patients with normolabuminuria (Ualbumin <30 mg/L). Shown are peptide sequences with start and stop amino acid position, corresponding protein ID (Swiss-Prot), peptide mass (Da) and migration time (min), the p-values (Mann-Withney U-test), mean amplitude in case and control group, and the regulation factor. Peptide modifications are denotd as: p = hydroxyproline; k = hydroxylysine; m = oxidized methionine).

Supplementary Table 2. Proteases predicted by Proteasix software based on the input list of 302 differentially expressed peptides. Proteases with the Status "Observed" or "Observed in different substrate..." (explained in "Methods") were retained for the activity score calculation and are displayed. Peptide modifications are denoted as: p = hydroxyproline; k = hydroxylysine; m = oxidized methionine. (Proteases with the Status "Predicted" i.e. with estimated probability of cleavage based on MEROPS protease specificity matrix, can be displayed by changing the filtering criteria in the "Status" column).

Supplementary Table 3. Protease activity score calculation for 30 experimentally observed proteases predicted by Proteasix tool. Scores were calculated as described in the Methods section. Positive scores indicate potentially activated proteases, while negative scores indicate deactivation.

Supplementary Table 4. Summary of DN datasets extracted from Nephroseq database.

Supplementary Table 5. Summary of 12 proteases reported in the transcriptomics datasets (Nephroseq, pval < 0.05) overlaping with the input of 17 predicted proteases. Proteases marked in red present inconsistent gene expression trend.

REFERENCES

1 Argilés, À. *et al.* CKD273, a New Proteomics Classifier Assessing CKD and Its Prognosis. *PLOS ONE* **8**, e62837, doi:10.1371/journal.pone.0062837 (2013).

2 Zurbig, P. *et al.* Biomarker discovery by CE-MS enables sequence analysis via MS/MS with platform-independent separation. *Electrophoresis* **27**, 2111-2125, doi:10.1002/elps.200500827 (2006).

3 Jantos-Siwy, J. *et al.* Quantitative urinary proteome analysis for biomarker evaluation in chronic kidney disease. *Journal of proteome research* **8**, 268-281, doi:10.1021/pr800401m (2009).

4 Good, D. M. *et al.* Naturally occurring human urinary peptides for use in diagnosis of chronic kidney disease. *Molecular & cellular proteomics : MCP* **9**, 2424-2437, doi:10.1074/mcp.M110.001917 (2010).

5 Neuhoff, N. *et al.* Mass spectrometry for the detection of differentially expressed proteins: a comparison of surface-enhanced laser desorption/ionization and capillary electrophoresis/mass spectrometry. *Rapid communications in mass spectrometry : RCM* **18**, 149-156, doi:10.1002/rcm.1294 (2004).

6 Klein, J., Papadopoulos, T., Mischak, H. & Mullen, W. Comparison of CE-MS/MS and LC-MS/MS sequencing demonstrates significant complementarity in natural peptide identification in human urine. *Electrophoresis* **35**, 1060-1064, doi:10.1002/elps.201300327 (2014).

7 Meleth, S., Deshane, J. & Kim, H. The case for well-conducted experiments to validate statistical protocols for 2D gels: different pre-processing = different lists of significant proteins. *BMC biotechnology* **5**, 7, doi:10.1186/1472-6750-5-7 (2005).

8 Coon, J. J. *et al.* CE-MS analysis of the human urinary proteome for biomarker discovery and disease diagnostics. *Proteomics. Clinical applications* **2**, 964, doi:10.1002/prca.200800024 (2008).

9 Siwy, J., Mullen, W., Golovko, I., Franke, J. & Zurbig, P. Human urinary peptide database for multiple disease biomarker discovery. *Proteomics Clin Appl* **5**, 367-374, doi:10.1002/prca.201000155 (2011).
